# Anti-infective activities of long-chain fatty acids against foodborne pathogens

**DOI:** 10.1093/femsre/fuad037

**Published:** 2023-07-12

**Authors:** Caroline Borreby, Eva Maria Sternkopf Lillebæk, Birgitte H Kallipolitis

**Affiliations:** Department of Biochemistry and Molecular Biology, University of Southern Denmark, Campusvej 55, 5230 Odense M, Denmark; Department of Biochemistry and Molecular Biology, University of Southern Denmark, Campusvej 55, 5230 Odense M, Denmark; Department of Biochemistry and Molecular Biology, University of Southern Denmark, Campusvej 55, 5230 Odense M, Denmark

**Keywords:** long-chain free fatty acids, foodborne pathogens, *Salmonella*, *Listeria monocytogenes*, antimicrobial activity, antivirulence activity

## Abstract

Free fatty acids (FFAs) have long been acknowledged for their antimicrobial activity. More recently, long-chain FFAs (>12 carbon atoms) are receiving increased attention for their potent antivirulence activity against pathogenic bacteria. In the gastrointestinal tract, foodborne pathogens encounter a variety of long-chain FFAs derived from the diet, metabolic activities of the gut microbiota, or the host. This review highlights the role of long-chain FFAs as signaling molecules acting to inhibit the infectious potential of important foodborne pathogens, including *Salmonella* and *Listeria monocytogenes*. Various long-chain FFAs interact with sensory proteins and transcriptional regulators controlling the expression of infection-relevant genes. Consequently, long-chain FFAs may act to disarm bacterial pathogens of their virulence factors. Understanding how foodborne pathogens sense and respond to long-chain FFAs may enable the design of new anti-infective approaches.

## Teaser

This review highlights the role of long-chain free fatty acids as signaling molecules acting to inhibit the infectious potential of important foodborne pathogens, including *Salmonella* and *Listeria monocytogenes*.

## Introduction

The concern over antibiotic resistance is increasing, and new strategies for combating bacterial infections are needed (Kwon and Powderly 2021). Free fatty acids (FFAs) have long been known for their antimicrobial activity, and FFAs are, therefore, considered as possible alternatives or complements to classical antibiotics (Desbois and Smith 2010, Yoon et al. 2018).

FFAs consist of a saturated or unsaturated carbon chain, and a terminal carboxy group (Table S1, Supporting Information). Since the carbon chain is hydrophobic and the carboxyl group is hydrophilic, FFAs are defined as amphipathic molecules holding the ability to drive spontaneous self-assembly (Yoon et al. 2018). The biological activity of FFAs depends on the length of the carbon chain, which ranges from short (< 8 carbon atoms) to medium (between 8 and 12 carbon atoms) and long (>12 carbon atoms). Furthermore, the number, position, and orientation (*cis* vs. *trans*) of double bonds determine the structure and functionality of unsaturated FFAs. In general, medium-chain saturated FFAs and long-chain unsaturated FFAs are the most active against bacteria, and Gram-positive species appear to be more susceptible to antimicrobial FFAs relative to Gram-negative species (Desbois and Smith 2010, Yoon et al. 2018). For long-chain unsaturated FFAs, the antimicrobial activity increases with length and degree of unsaturation. Reported minimal inhibitory concentrations (MIC values) of medium- and long-chain FFAs often differ between studies due to variations in experimental conditions (i.e. growth medium, temperature, and pH) (Yoon et al. 2018). Furthermore, the use of different organic solvents for solubilizing the antimicrobial FFAs likely affects their concentration-dependent molecular self-assembly (Yoon et al. 2018).

In biological systems, FFAs are typically bound to other compounds to form lipids, e.g. triglycerides in dietary fat, or phospholipids in membranes, but they can be released as FFAs by the enzymatic activities of lipases. Since FFAs serve as nutrient sources and are vital for building membranes, bacteria willingly take up FFAs from the environment (Yao and Rock 2017). Inside the cytosol, exogenous FFAs are activated in an organism-specific manner and may then be degraded to generate energy or serve as membrane building blocks. In the absence of exogenous FFAs, bacteria must synthesize these building blocks to sustain growth (Radka and Rock 2022). The bacterial fatty acid biosynthesis type II system (FASII) performs all steps required for *de novo* synthesis of fatty acids, and FASII enzymes are, therefore, essential for growth in environments devoid of FFAs.

FFAs are used as antimicrobial agents by many organisms to defend themselves against microbial pathogens. The human skin, respiratory tract, and gastrointestinal tract represent three major environments, where bacterial pathogens encounter antimicrobial FFAs produced by the host or resident microbiota (Fischer 2020, Metzler-Zebeli et al. 2021). Due to their amphipathic nature, medium- and long-chain FFAs with antimicrobial properties are thought to target and accumulate within the bacterial membrane (Desbois and Smith 2010, Yoon et al. 2018). High doses of antimicrobial FFAs may destabilize the bacterial membrane, leading to increased cell permeability and cell lysis. Furthermore, FFAs may interfere with essential biological processes in the membrane, such as energy production and nutrient uptake, in direct or indirect ways. Importantly, bacteria may sense the presence of FFAs and build up protective responses against their toxic activities (Desbois and Smith 2010, Kengmo Tchoupa et al. 2022). To defend themselves against FFAs, bacteria may produce a more protective cell surface to prevent FFAs from reaching the cell membrane. Furthermore, some pathogens are known to encode efflux systems acting to export FFAs from the cell, or detoxification factors that inactivate antimicrobial FFAs.

Although resistance development potentially could limit the use of FFAs as antimicrobial agents in the future, recent findings indicate that they may be used in alternative ways to combat pathogenic bacteria. At low doses, medium- and long-chain FFAs act as signaling compounds controlling important traits of bacterial pathogens, such as biofilm formation and virulence (Kumar et al. 2020, Mirzaei et al. 2022, Mitchell and Ellermann 2022). Specific FFAs modulate the activity of sensory proteins and transcriptional regulators to suppress the expression of key virulence genes required for establishing a successful infection. Notably, medium- and long-chain FFAs without antimicrobial activity may act to reduce the virulence of a pathogen, suggesting that some FFAs hold potential as anti-infective agents without affecting the growth and survival of the resident microbiota.

This review will focus on how long-chain FFAs inhibit the growth and/or virulence-potential of foodborne pathogens. Specifically, we wish to address how this knowledge could be used against important foodborne pathogens in the future. To illustrate some of the central findings, we will focus on two major foodborne pathogens, the Gram-negative bacterium *Salmonella enterica* serovar Typhimurium, and the Gram-positive bacterium *Listeria monocytogenes*. For detailed information on how long-chain FFAs affect other bacterial pathogens (e.g. *Staphylococcus aureus* and *Pseudomonas aeruginosa*), we refer to recent reviews covering these organisms (Kumar et al. 2020, Kengmo Tchoupa et al. 2022).

## Long-chain FFAs acting against foodborne pathogens

Foodborne pathogens encounter a mixture of saturated, monounsaturated, and polyunsaturated long-chain fatty acids in food products and during passage of the gastrointestinal tract. Animal fat is the most common source for saturated fatty acids, whereas unsaturated fatty acids are found in high amounts in fish, nuts, and vegetable oils. Meat fats contain about 40% saturated fatty acids, mainly palmitic acid (C16:0) and stearic acid (C18:0), however, the major fatty acid in meats is monounsaturated oleic acid (C18:1), contributing over 30% of total fatty acids (Wood et al. 2008) (Table S1, Supporting Information). Bovine milk fat contains a high proportion of saturated fatty acids (around 70%) as well as monounsaturated fatty acids (around 25%), mainly palmitic acid (C16:0) and oleic acid (C18:1), respectively (Jensen et al. 1991, Mansson 2008). Oils from olives and hazelnuts contain high amounts of oleic acid (C18:1) (around 70%), whereas walnuts are known for their high content of polyunsaturated omega-6 fatty acid linoleic acid (C18:2) (Yorulmaz et al. 2013, Monika and Anna 2019) (Table S1, Supporting Information). Fish oils are particularly rich in the long-chain polyunsaturated omega-3 fatty acids eicosapentaenoic acid (C20:5) and docosahexaenoic acid (C22:6) (Ward et al. 2022) (Table S1, Supporting Information).

Importantly, most fatty acids in food are bound to glycerol in the form of triglycerides, which are not active against bacteria. Thus, foodborne pathogens are not expected to encounter high levels of freely available long-chain FFAs until they reach the gastrointestinal tract (Chadaideh and Carmody 2021), unless they reside in fermented or partially spoiled foods, where lipases produced by other microorganisms (e.g. *Pseudomonas* spp.) catalyze the release of long-chain FFAs from triglycerides in the foods (Arslan et al. 2011). In the intestine, dietary fat is emulsified by the actions of bile salts and FFAs are liberated from the triglycerides through the enzymatic actions of pancreatic lipase. Long-chain FFAs are predominantly incorporated into bile salt-mixed micelles, which increases the water solubility of the FFAs, thus priming them for uptake (Murota and Storch 2005). Then, FFAs are absorbed by the epithelial cells of the small intestine. Dietary fat affects the composition and function of the natural microbial inhabitants of the gut, and the gut microbes contribute to the overall fatty acid metabolism in the gastrointestinal tract (Agans et al. 2018, Martinez-Guryn et al. 2018, Saika et al. 2019, Chadaideh and Carmody 2021, Mirzaei et al. 2022). The gut microbiota is well known to produce short-chain fatty acids (SCFA), with important roles as anticarcinogenic and anti-inflammatory agents. Moreover, SCFAs act to modulate the expression of bacterial virulence factors. For an overview on how SCFAs influence foodborne pathogens, we refer to a recent review covering this topic (Mirzaei et al. 2022).

Long-chain fatty acids produced by the host and intestinal bacteria play important roles in the regulation of human health and diseases. Conjugated linoleic acid (CLA), which is an isomer of linoleic acid (C18:2) with conjugated double bonds, is produced by enzymatic activities of the gut microbiota (O’Shea et al. 2012, Salsinha et al. 2018) (Table S1, Supporting Information). CLA has gained significant attention due to its wide range of potential health benefits, including anticarcinogenic, antiobese, antidiabetic, and anti-inflammatory effects (Koba and Yanagita 2014, Saika et al. 2019). Notably, CLA also exerts anti-infective activities against some enteric pathogens. Recent studies suggest that unknown members of the gut microbiota produce a *cis*-2 unsaturated long-chain FFA, which belongs to a family of diffusible signal factors (DSF) (He et al. 2023) (Table S1, Supporting Information). Interestingly, specific DSFs have been found to inhibit the production of virulence factors in *Salmonella* and *Vibrio cholerae*. In line with this, several unsaturated long-chain FFAs present in bile, such as palmitoleic acid (C16:1) and oleic acid (C18:1), are known to affect the virulence-potential of enteric pathogens (Table S1, Supporting Information) (Chatterjee et al. 2007, Golubeva et al. 2016).

Altogether, foodborne pathogens are expected to encounter a variety of biologically active long-chain fatty acids derived from the diet, host, and gut microbiota in the gastrointestinal tract. Although the concentrations and bioavailability of long-chain FFAs in the human gut remain unknown, recent studies in pigs revealed that the fatty acids profiles vary along the gastrointestinal tract, with long-chain FFAs found at higher levels in the stomach, cecum, and colon, relative to the jejunum and ileum of the small intestine (Metzler-Zebeli et al. 2021, Mitchell and Ellermann 2022). Thus, foodborne pathogens may use long-chain FFAs as signaling molecules to establish a successful infection at the right time and place during intestinal passage. In the following sections, examples of how *Salmonella, L. monocytogenes*, and related bacteria sense and respond to long-chain FFAs will be presented.

## Salmonella

### Long-chain FFAs inhibit the activity of AraC-family regulators in *Salmonella* and related Gram-negative pathogens

The Gram-negative pathogen *S*. Typhimurium is the causative agent of both acute gastroenteritis and systemic diseases (Menard et al. 2022). *Salmonella* infections are often associated with ingestion of contaminated food products, such as eggs and chicken, or consumption of water contaminated with feces from infected hosts (Velge et al. 2012). At the initial stages of infection, *Salmonella* invades the intestinal epithelial cells using a type III section system (T3SS) encoded by the *Salmonella* pathogenicity island-1 (SPI-1) (Patel and Galan 2005). The needle-like T3SS apparatus injects bacterial effector proteins into the cytosol of the infected host cells, leading to rearrangements of the actin cytoskeleton and activation of inflammatory pathways. A second *Salmonella* pathogenicity island, SPI-2, encodes another T3SS system, which is required for intracellular growth and survival of this pathogen (Jennings et al. 2017). The SPI-2 T3SS translocates a broad range of effector proteins with diverse biochemical functions from *Salmonella*-containing vacuoles into the host cells.

Medium-length saturated FFAs, such as caprylic acid (C8:0) and lauric acid (C12:0) (Table S1, Supporting Information), show growth-inhibitory activity against *Salmonella* species, whereas the antimicrobial activity of long-chain FFAs is limited against these Gram-negative bacteria (Petschow et al. 1998, Skrivanova et al. 2004, Van Immerseel et al. 2004, Shin et al. 2007). Although *S*. Typhimurium grows well in the presence of high levels of long-chain FFAs, they do not leave this pathogen unaffected. Specific long-chain FFAs act as signaling molecules that modulate expression of virulence genes in *S*. Typhimurium by affecting the activities of key virulence regulators of SPI-1, SPI-2, and other virulence-relevant genes (Viarengo et al. 2013, Golubeva et al. 2016). In the following, the effect of long-chain FFAs on AraC-like transcriptional regulators controlling SPI-1 will be addressed.

**Figure 1.**
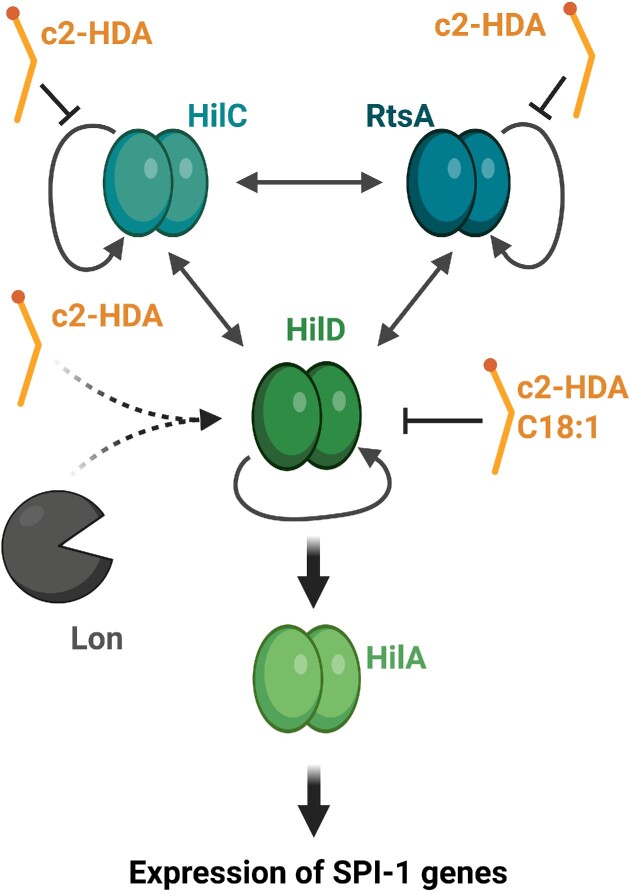
Long-chain FFAs inhibit the expression of SPI-1 virulence genes in *Salmonella*. The regulatory proteins HilD, HilC, and RtsA form a feed-forward loop controlling the virulence regulator HilA, which activates expression of SPI-1 genes. Long-chain FFAs, such as oleic acid (C18:1) and the DSF compound c2-HDA (Table S1, Supporting Information), interfere with the regulatory activity of HilD. Furthermore, c2-HDA promotes Lon-mediated degradation of HilD and inhibits the regulatory activities of HilC and RtsA.

The genes encoding T3SS from SPI-1 are controlled by the transcriptional regulator HilA (Bajaj et al. 1995, Eichelberg and Galan 1999). This regulator is positively controlled by three other regulators, HilD, HilC, and RtsA; together, these regulators form a feed-forward loop controlling the expression of SPI-1 T3SS (Ellermeier et al. 2005) (Fig. 1). Interestingly, exposure to saturated or unsaturated long-chain FFAs down-regulates transcription of *hilA*, leading to decreased expression of SPI-1 genes (Golubeva et al. 2016). Specifically, oleic acid (C18:1), myristic acid (C14:0) (Table S1, Supporting Information), and palmitic acid (C16:0) exert an inhibitory effect on *hilA* transcription. *Salmonella* Typhimurium can take up and use long-chain FFAs as an energy source, but degradation of these FFAs is not required for repression of virulence genes (Golubeva et al. 2016). Indeed, mouse infection experiments with mutant strains defective in the uptake and/or metabolism of long-chain FFAs showed that *Salmonella* primarily uses long-chain FFAs as signaling compounds to dictate the expression of T3SS at the right time and place. Regarding the mechanism underlying the antivirulence activity of long-chain unsaturated FFAs, experiments with oleic acid (C18:1) revealed that this FFA acts directly on HilD to prevent the regulator from binding to DNA (Golubeva et al. 2016) (Fig. 1). Consequently, transcription of *hilA* is suppressed in response to long-chain unsaturated FFAs, leading to reduced expression of T3SS from SPI-1.

**Figure 2.**
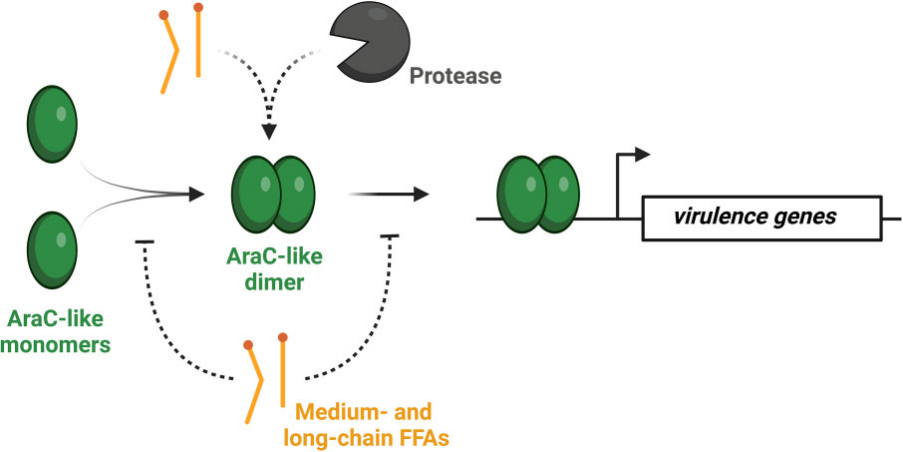
Model of how medium- and long-chain FFAs interfere with AraC-like transcriptional regulators. AraC family members, such as HilD in *Salmonella*, ToxT in *V. cholerae*, and Rns in EHEC, are known to activate the expression of virulence genes. Specific medium- and/or long-chain FFAs inhibit the stability, dimerization and/or DNA-binding activity of AraC-like regulators.

HilD belongs to the AraC-family of proteins, and other members of this protein family have been shown to respond directly to long-chain FFAs. The best studied example is the virulence regulator ToxT in *V. cholerae*. ToxT activates transcription of *ctxAB* and *tcpA* encoding cholera toxin and the toxin-coregulated pilus, which are required for *V. cholerae* to cause disease (Peterson and Gellings 2018). The ToxT regulator activates transcription by binding as a monomer or dimer to specific tox-boxes located in the promoter regions of ToxT-activated genes (Withey and DiRita 2006). Initially, experiments were conducted with crude bile to identify components responsible for repression of virulence genes in *V. cholerae* (Chatterjee et al. 2007). The expression of cholera toxin was found to be efficiently repressed by the fatty acid-containing component of bile, and five individual long-chain FFAs were isolated from this fraction for further analyses. The long-chain unsaturated FFAs arachidonic acid (C20:4) (Table S1, Supporting Information), linoleic acid (C18:2), and oleic acid (C18:1) were found to inhibit cholera toxin production, whereas the long-chain saturated FFAs palmitic acid (C16:0) and stearic acid (C18:0) had no effect. Notably, high levels of the three unsaturated long-chain FFAs had a growth-inhibitory effect as well on *V. cholerae* (Chatterjee et al. 2007). In later studies, structural analyses of ToxT revealed that palmitoleic acid (C16:1) was tightly bound to a ToxT monomer (Lowden et al. 2010). Palmitoleic acid (C16:1), oleic acid (C18:1), and linoleic acid (C18:2) were found to inhibit transcription of *tcp* and *ctxAB*, and they prevented ToxT from binding to the promoter regions of virulence genes (Lowden et al. 2010, Plecha and Withey 2015). In contrast, the saturated FFA palmitic (C16:0) had no effect on the regulatory and DNA-binding activity of ToxT (Lowden et al. 2010). Further structural and mutational studies supported a model where FFA-free ToxT adopts an active conformation that can dimerize and bind DNA, whereas binding of unsaturated long-chain FFAs to ToxT results in an inactive conformation that precludes dimerization and binding to DNA (Childers et al. 2011, Cruite et al. 2019). Altogether, these studies on ToxT illustrate how unsaturated long-chain FFAs act to inhibit the DNA-binding activity of AraC-family members (Fig. 2).

Since linoleic acid (C18:2) is a potent inhibitor of ToxT activity, the antivirulent effect of commercially acquired CLA was investigated in *V. cholerae* (Withey et al. 2015). CLA was found to inhibit the production of cholera toxin and toxin-coregulated pilus *in vitro*, and it reduced the DNA-binding activity of ToxT. Interestingly, administration of CLA reduced the production of cholera toxin in a rabbit ileal loop model for cholera disease. Furthermore, cholera toxin-induced fluid accumulation was reduced upon administration of CLA *in vivo* (Withey et al. 2015). These findings suggest that CLA could be used as a therapeutic against cholera.

Enterohemorrhagic *Eschericia coli* (EHEC) is known to cause severe diarrhea upon consumption of contaminated foods (Khalil et al. 2018). Gut colonization by EHEC relies on the production of a variety of adhesive pili, and their expression is controlled by the AraC family member Rns (Munson 2013). Structural studies on Rns revealed the presence of the medium-chain saturated FFA decanoic acid (C10:0) (Table S1, Supporting Information) bound in a pocket within Rns (Midgett et al. 2021). Importantly, exposure to decanoic acid (C10:0) inhibits Rns-dependent expression of pili in EHEC. The structural mechanism by which Rns is regulated by decanoic acid (C10:0) remains to be clarified, but this study supports that medium-chain saturated FFAs also act to control virulence gene expression in enteric pathogens by binding directly to an AraC family member (Fig. 2). Interestingly, it should be noted that the medium-chain saturated FFA caprylic acid (C8:0) exerts dual effects on *Salmonella*. As mentioned above, caprylic acid (C8:0) shows growth-inhibitory activity against *Salmonella*, but at subinhibitory concentrations, this fatty acid acts to reduce transcription of *hilA* (Van Immerseel et al. 2004). The mechanism underlying the inhibitory effect of caprylic acid (C8:0) on *hilA* expression remains to be uncovered. Clearly, future studies should address whether medium-chain saturated FFAs affect the activity of the three AraC-like regulators controlling the expression of *hilA*.

Recent findings further substantiate a role for long-chain unsaturated FFAs in controlling the activity of regulators of the AraC family in enteric bacteria. A search for chemicals acting to inhibit invasion gene expression in *S*. Typhimurium resulted in the identification of fatty acids belonging to the DSF-class of quorum sensing signaling molecules (Bosire et al. 2020). DSF molecules are *cis*-2 unsaturated fatty acids produced by Gram-negative bacteria, and they are known to control important biological processes in plant pathogens, including biofilm formation and virulence (Kumar et al. 2020, He et al. 2023). The DSF compound *cis*-2-hexadecenoic acid (c2-HDA) has a chain length of 16 carbons with a single *cis* unsaturation at the second carbon (Table S1, Supporting Information). Interestingly, c2-HDA acts to inhibit the expression of SPI-1 virulence genes *hilA* and *sopB* in *S*. Typhimurium and the cholera toxin-encoding genes *ctxAB* in *V. cholerae* (Bosire et al. 2020). Indeed, c2-HDA is a more efficient inhibitor of virulence gene expression in *S*. Typhimurium than the unsaturated long-chain FFA oleic acid (C18:1) from bile. In *Salmonella*, HilD is the primary target for c2-HDA activity, and the DSF-compound prevents HilD from binding to DNA. Notably, c2-HDA also acts to destabilize the HilD protein via Lon protease activity (Bosire et al. 2020, Chowdhury et al. 2021b) (Fig. 1). In comparison, oleic acid (C18:1) inhibits the DNA-binding activity of HilD, but this FFA does not affect the stability of HilD. Exposure to c2-HDA, as well as oleic acid (C18:1), reduces invasion of epithelial cells, suggesting that long-chain unsaturated FFAs could act to prevent *Salmonella* from causing intestinal infections.

So far, no dietary sources for *cis*-2-unsaturated fatty acids are known, but a recent study suggests that *Salmonella* and other intestinal pathogens may well encounter DSF compounds in the gut (Chowdhury et al. 2021b). More specifically, c2-HDA could be extracted from the large intestinal content of mice, showing that this signaling compound indeed is present in the mammalian intestine. Structural and mutational studies identified different residues of HilD required for repression by various types of long-chain FFAs. For c2-HDA, the carboxylate head group as well as the *cis*-2 unsaturation are important for recognition by HilD. Notably, c2-HDA readily outcompetes other intestinal long-chain fatty acids for HilD-mediated repression of invasion, suggesting that *Salmonella* uses this specific signal over other fatty acids to control invasion in the intestinal environment (Chowdhury et al. 2021b). Intriguingly, recent findings show that the AraC-like virulence regulators HilC and RtsA respond to the presence of c2-HDA as well (Chowdhury et al. 2021a) (Fig. 1). As mentioned above, the HilC and RtsA regulators, together with HilD, ensure complete activation of SPI-1 by activating transcription of each other as well as *hilA*. Indeed, c2-HDA can bind directly to all three regulators, perturbing their interaction with *hilA* promoter DNA, but in contrast to HilD, the stability of HilC and RtsA is not affected by c2-HDA (Chowdhury et al. 2021a, b). Since HilC and RtsA are not degraded, HilD protein levels are rapidly restored when c2-HDA signaling ends, leading to induction of SPI-1 expression. HilC and RtsA are generally less responsive to c2-HDA compared to HilD, enabling *Salmonella* to regain virulence more quickly after removal of c2-HDA (Chowdhury et al. 2021a).

Altogether, these findings demonstrate that c2-HDA and other long-chain FFAs act as signaling molecules to modulate bacterial virulence by direct binding to AraC-like transcription activators (Fig. 2). The FFAs encountered by foodborne pathogens during passage of the intestinal tract may be derived from food sources, by activities of the host or the resident microbiota. However, the specific origin of the most potent fatty acid inhibitor of *Salmonella* virulence, c2-HDA, is not yet known. DSF compounds, including c2-HDA, are most likely produced by Gram-negative species of the intestinal microbiota, serving as one of the many signals sensed by *Salmonella* and other enteric pathogens during intestinal passage (He et al. 2023). Ultimately, these signaling molecules determine where and when *Salmonella* chooses to initiate the infection program encoded from SPI-1. Intriguingly, a recent study found that recombinant production of c2-HDA by *E. coli* in the gut of chickens could inhibit *Salmonella* invasion and animal carriage (Rather et al. 2023). Further investigations are required to evaluate the practical use of *in situ* production of c2-HDA to reduce the carriage of *Salmonella* in production animals (Rather et al. 2023).

### Long-chain FFAs affect the activity of the two-component system PhoP/PhoQ in *Salmonella*

**Figure 3.**
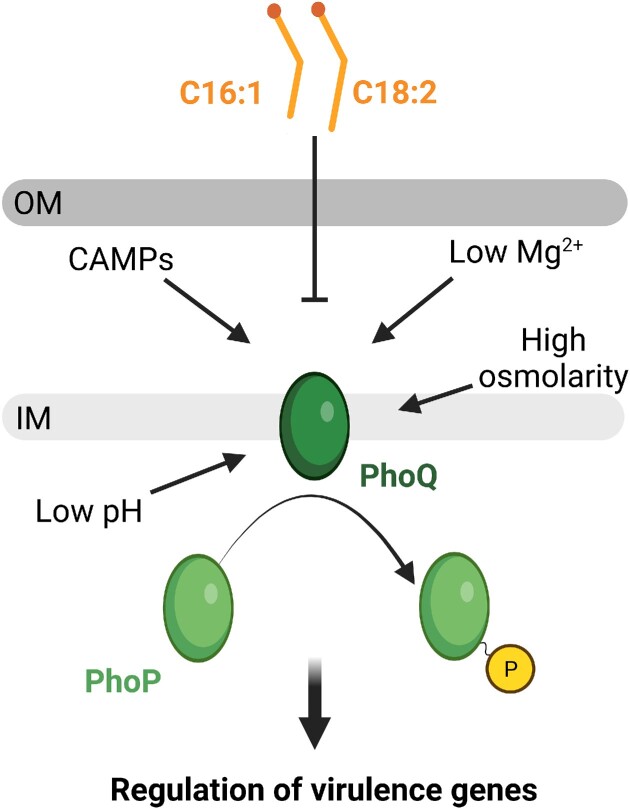
Long-chain unsaturated FFAs inhibit the PhoP/PhoQ two-component system in *Salmonella*. The histidine kinase PhoQ autophosphorylates in the presence of an activating signal, such as Mg^2+^ limitation, cationic antimicrobial peptides (CAMPs), low pH, and high osmolarity. The phosphoryl group is transferred to the response regulator PhoP, which acts as a transcription regulator of virulence genes. Long-chain unsaturated FFAs, such as palmitoleic acid (C16:1) and linoleic acid (C18:2) (Table S1, Supporting Information), interfere with the autokinase activity of PhoQ, thereby preventing phosphorylation of the cytoplasmic response regulator PhoP. Consequently, PhoP-dependent regulation of virulence genes is repressed. OM: outer membrane. IM: inner membrane.

The two-component system PhoP/PhoQ plays a major role in controlling the expression of virulence-related genes in *Salmonella*, including SPI-1 and SPI-2 genes (Groisman et al. 2021). In the presence of an activating signal, the transmembrane sensor PhoQ autophosphorylates, after which the phosphoryl group is transferred to the cytoplasmic response regulator PhoP. Phosphorylated PhoP exerts its role as transcriptional regulator by binding to specific sequences in the promoter regions of PhoP/PhoQ regulated genes (Zwir et al. 2012). In the absence of an activating signal, PhoQ dephosphorylates PhoP, which attenuates its transcriptional regulator activity. Among the PhoQ activating conditions are Mg^2+^ limitation, specific cationic antimicrobial peptides, low pH, and high osmolarity (Garcia Vescovi et al. 1996, Bader et al. 2005, Prost et al. 2007, Yuan et al. 2017) (Fig. 3). Intriguingly, a screen for molecules from plant extracts that would modulate PhoP/PhoQ activity led to the identification of unsaturated long-chain FFAs as inhibitory input signals (Viarengo et al. 2013). More specifically, unsaturated long-chain FFAs of the C16 and C18 series were found to reduce the expression of genes activated by PhoP, whereas the saturated long-chain FFAs palmitic acid (C16:0) and stearic acid (C18:0) had no effect on PhoP-dependent activation of transcription. The inhibitory effect does not rely on cellular uptake of fatty acids or a functional metabolic β-oxidative pathway, demonstrating that exogeneous unsaturated long-chain FFAs are sensed directly by the two-component system (Viarengo et al. 2013) (Fig. 3). Indeed, C18:2 FFAs were shown to inhibit the autokinase activity of PhoQ by a mechanism that involves a conformational change in the periplasmic sensor domain (Carabajal et al. 2020). Accordingly, PhoP-dependent regulation of gene expression is repressed upon exposure to these unsaturated long-chain FFAs.

CLA, corresponding to conjugated C18:2, inhibits PhoQ autokinase activity as well, and the effect of oral administration of CLA on *Salmonella*-induced colitis in mice was, therefore, tested (Carabajal et al. 2020). Notably, the animals were pretreated with streptomycin to provide a more robust model for *Salmonella*-induced colitis. In this model, *phoP* primarily contributes to the later stages of infection, where *Salmonella* reaches the spleen (Carabajal et al. 2020). Curiously, mice treated with CLA turned out to be more susceptible to *Salmonella* infection compared to untreated controls, most likely reflecting the complex interplay between bacterial and host responses when exposed to dietary CLA supplementation.

### Long-chain FFAs control the activity of FadR in *Salmonella* and related Gram-negative pathogens

In Gram-negative enteric pathogens, the metabolism of exogenous long-chain FFAs has been linked to infection via the regulatory actions of the GntR/TetR family regulator, FadR (Pifer et al. 2018, Ellermann et al. 2021). When long-chain FFAs are taken up from the environment, they are esterified in the cytoplasm into long-chain acyl coenzyme A (CoA) thioesters (Fujita et al. 2007). Long-chain acyl-CoAs serve as substrates for membrane biosynthesis or energy production, and they are directly sensed by the FadR regulator. FadR binds the CoA moiety of the activated fatty acids, resulting in a decreased affinity for DNA (van Aalten et al. 2000). In its unbound form, FadR binds to DNA and activates the *fab* genes encoding the fatty acid biosynthesis pathway, whereas the fatty acid degradation machinery, encoded by the *fad* genes, is under negative control by FadR (Fujita et al. 2007). During the intestinal phase of infection, the regulatory activities of FadR contribute to gut colonization of *Salmonella* (Hoshino et al. 2022). More specifically, FadR affects the expression of genes involved in flagellar motility, and proper flagellar motility is required for colonization of the gut. Notably, fatty acid metabolism by β-oxidation seems to be dispensable during colonization of the gut, supporting that long-chain FFAs are primarily used by *Salmonella* as a signaling molecule, rather than an energy source in the gut (Hoshino et al. 2022). However, during the systemic phase of infection, fatty acid metabolism appears to be required for tissue colonization (Reens et al. 2019).

FadR was recently shown to play an important role in virulence gene expression in EHEC (Pifer et al. 2018, Ellermann et al. 2021). FadR is an activator of genes belonging to the locus of enterocyte effacement (LEE) pathogenicity island encoding a type 3 secretion system (T3SS). The T3SS injects effector molecules into colonic epithelial cells, where they promote cytoskeletal rearrangements known as the attaching and effacing (A/E) lesions characteristic for EHEC disease. Upon exposure to long-chain fatty acids, including palmitic acid (C16:0) and arachidonic acid (C20:4), the FFAs are taken up by EHEC and converted into acyl-CoA derivatives. Next, FadR-dependent regulation of LEE is modulated by direct binding of the long-chain acyl-CoAs to FadR, leading to decreased binding of FadR to its DNA motifs and, consequently, inhibition of EHEC virulence. Indeed, treatment of EHEC with arachidonic acid (C20:4) prevents this pathogen from forming the characteristic A/E lesions on epithelial cells in a FadR-dependent manner (Ellermann et al. 2021).

Interestingly, FadR indirectly stimulates expression of the virulence regulator ToxT in the El Tor biotype of *V. cholerae*, through two different mechanisms (Kovacikova et al. 2017). First, loss of FadR reduces the transcription of *toxT* in an indirect manner, and second, inactivation of *fadR* reduces the levels of ToxT post-translationally. These findings suggest that FadR constitutes a regulatory link between fatty acid metabolism and virulence in specific *V. cholerae* biotypes (Kovacikova et al. 2017). Notably, no role for FadR in virulence regulation is observed in the classical biotype of *V. cholerae* (Ray et al. 2011).

### Dietary supplementation of omega-3 fatty acids affects the course of *Salmonella* infection

Dietary fish oil rich in omega-3 long-chain polyunsaturated fatty acids is well known for its various health beneficial effects, and omega-3 fatty acids are widely recommended for treating chronic inflammatory diseases, heart diseases, type 2 diabetes and autoimmune diseases, amongst others (Husson et al. 2016). More specifically, long-chain polyunsaturated FFAs serve as precursors for eicosanoids, which play important roles as signaling molecules in diverse biological processes, including immunomodulatory roles (Sheppe and Edelmann 2021). In the past, mouse infection studies demonstrated that a diet rich in fish oil for 4 weeks decreases survival after peroral challenge with *S*. Typhimurium (Chang et al. 1992). In line with this, the bacterial counts in the liver and spleen were higher in mice fed on fish oil diet compared to mice fed on a low-fat diet, following intraperitoneal infection to mimic a systemic infection (Chang et al. 1992). In contrast, a more recent study reported a protective effect of the long-chain polyunsaturated omega-3 fatty acids eicosapentaenoic acid (C20:5) and docosahexaenoic acid (C22:6) against *Salmonella* infection (Liu et al. 2020). Mice fed with an omega-3 enriched diet for 7 days before infection with S. *Typhimurium* demonstrated an increased survival relative to mice fed with an omega-6 enriched diet or a regular diet. *Salmonella* was found to be phagocytized more efficiently by macrophages in the presence of the omega-3 FFAs, and the intestinal production of SCFAs with antibacterial and anti-inflammatory properties was significantly increased. In line with this, the composition of the intestinal microbiota was changed in a direction that likely promotes host resistance towards *Salmonella* infection. Notably, growth of *Salmonella* is not inhibited by the omega-3 FFAs, and virulence gene expression is unaffected in the presence of omega-3 FFAs (Liu et al. 2020). These findings suggest that the long-chain polyunsaturated omega-3 FFAs protects against *Salmonella* infections by inducing beneficial changes to gut microbiota and host immune responses.

Differences in the experimental setup and methodological analyses (e.g. 4 weeks of feeding on a fish oil diet vs. 7 days on a diet enriched in omega-3 FFAs) may well explain the discrepancies observed between results of the mouse infection studies (Chang et al. 1992, Liu et al. 2020). Clearly, more research on long-chain polyunsaturated FFAs and eicosanoid functions are required to fully understand their roles in the course of bacterial infections (Sheppe and Edelmann 2021).

## Listeria monocytogenes

### Long-chain FFAs exert antimicrobial activity against *L. monocytogenes* and related intestinal Gram-positive bacteria

*Listeria monocytogenes* can be found in a variety of food-processing environments and food products, and infections by this Gram-positive pathogen are commonly associated with the consumption of ready-to-eat foods (Kallipolitis et al. 2020, Koopmans et al. 2023). *Listeria monocytogenes* is well known for its ability to grow and survive under a wide range of stressful environmental conditions, including stress factors encountered along the food chain, such as acidic pH, osmotic stress, and low temperatures (Bucur et al. 2018). After consumption of contaminated foods, *L. monocytogenes* enters the gastrointestinal tract leading to gastroenteritis in otherwise healthy individuals. *Listeria monocytogenes* can cross the intestinal barrier and cause life-threatening conditions such as meningitis and encephalitis in susceptible individuals, or still-birth and spontaneous abortion in pregnant women (Freitag et al. 2009, Koopmans et al. 2023).

*Listeria monocytogenes* has been found in dairy foods and several studies in the past focused on elucidating the antilisterial effect of fatty acids typical for milk (Wang and Johnson 1992, Petrone et al. 1998, Sprong et al. 2001). They found that medium-chain saturated FFAs, such as lauric acid (C12:0), are most active against *L. monocytogenes*, together with long-chain unsaturated FFAs of the C18 series. *Listeria monocytogenes* is generally sensitive towards mono- and polyunsaturated long-chain FFAs, including the omega-3 FFA eicosapentaenoic acid (C20:5) (Shin et al. 2007, Sternkopf Lillebaek et al. 2017, Chen et al. 2021). The specific mechanisms by which medium- and long-chain FFAs act against *L. monocytogenes* is presently unclear, but electron microscopic analyses of FFA-treated cells revealed a filamentous appearance and slightly irregular cell surface, supporting that the membrane is the primary target for antimicrobial medium- and long-chain FFAs (Wang and Johnson 1992).

**Figure 4.**
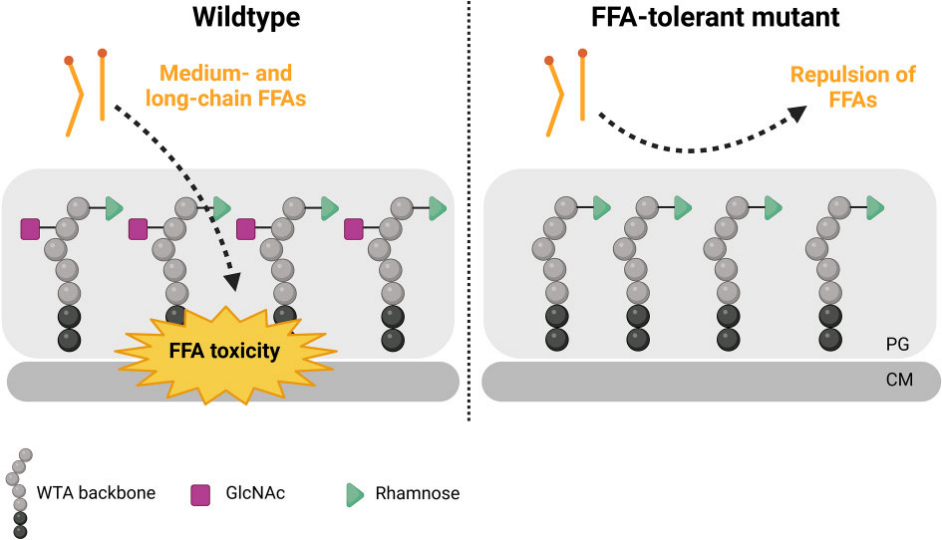
Lack of GlcNAc glycosylation on WTAs promotes FFA tolerance in *L. monocytogenes*. In the wildtype situation, *L. monocytogenes* serotype 1/2a contains two modifications of the WTAs: GlcNAc and rhamnose. Antimicrobial medium- and long-chain FFAs are expected to target the bacterial membrane (left). FFA-tolerant strains lacking GlcNAc glycosylation on WTAs are characterized by a more hydrophilic cell surface and reduced binding of FFAs (right). Consequently, the FFAs are repulsed from the bacterial surface, thereby protecting the mutant strain from FFA toxicity. PG: peptidoglycan layer. CM: cytoplasmic membrane.

Not surprisingly, bacteria are capable of developing resistance against the antimicrobial activity of FFAs. Indeed, FFA-tolerant strains were readily isolated by serial passage of *L. monocytogenes* in broth with increasing concentrations of antimicrobial medium- and long-chain FFAs (Thomasen et al. 2022a, b). Genome sequencing of FFA-tolerant isolates revealed mutations in genes involved in glycosylation of wall teichoic acids (WTAs) (Thomasen et al. 2022a). Lack of *N*-acetylglucosamine (GlcNAc) glycosylation on WTAs allows growth in the presence of high levels of lauric acid (C12:0), palmitoleic acid (C16:1), and eicosapentaenoic acid (C20:5) (Thomasen et al. 2022a). Furthermore, absence of GlcNAc slightly increase the sensitivity toward the antimicrobial peptide CRAMP, and WTA glycosylations are known to confer decreased susceptibility toward gentamicin and ampicillin (Meireles et al. 2020). The FFA-tolerant isolates are characterized by a more hydrophilic cell surface and reduced binding of FFAs to the bacterial surface (Thomasen et al. 2022a). Most likely, these mutants are better protected from antimicrobial FFAs, because the FFAs are repulsed from their surface (Fig. 4). A different subset of FFA-tolerant isolates was found to carry mutations in the *ccpA* gene encoding the catabolite control protein A, CcpA, a major transcriptional regulator of carbon catabolite repression in *L. monocytogenes* (Thomasen et al. 2022b). Inactivation of *ccpA* promotes growth in the presence of lauric acid (C12:0) and palmitic acid (C16:1) and results in a more hydrophilic cell surface. Notably, mutations conferring tolerance toward antimicrobial medium- and long-chain FFAs also support growth of *L. monocytogenes* at low pH (Thomasen et al. 2022a, b).

Gram-positive bacteria of the gut microbiota are affected by long-chain FFAs as well. For species of the genus *Lactobacillus*, growth in synthetic media is stimulated by the addition of Tween 80, which is rich in oleic acid (C18:1); furthermore, this growth supplement enhances survival of some Lactobacilli under acidic conditions, most likely due to the incorporation of exogenous long-chain FFAs in the bacterial membrane (Corcoran et al. 2007). Interestingly, some beneficial *Lactobacillus* species are inhibited by long-chain unsaturated FFAs linoleic acid (C18:2), oleic acid (C18:1), and α-linolenic acid (C18:3) (Table S1, Supporting Information) (Di Rienzi et al. 2018), which are commonly found in vegetable oil from soybeans (Jiang et al. 1998, Kankaanpaa et al. 2001, Jenkins and Courtney 2003). The consumption of soybean oil in the Western world has increased dramatically during the past century. During the same period, the prevalence of beneficial Lactobacilli has declined in Western gut microbiomes (Walter et al. 2011). The beneficial Gram-positive bacteria *Lactobacillus reuteri* and *Lactobacillus johnsonii* still exist in people who consume a Western diet, suggesting that they are protected from antimicrobial FFAs in the gut, or that they become resistant to these compounds (Di Rienzi et al. 2018). *In vitro* evolution experiments demonstrated that *L. reuteri* and *L. johnsonii* become resistant to the toxic effects of linoleic acid (C18:2) through mutations in genes related to fatty acid metabolism, cell wall and membrane, and resistance to acid stress (Di Rienzi et al. 2018). The Lactobacilli persisted through both chronic and acute exposures to linoleic acid (C18:2) in the mouse gut. Interestingly, FFA-resistant *L. reuteri* isolates were retrieved from the small intestine of mice given a high-fat diet, supporting that chronic exposure to a diet high in linoleic acid (C18:2) promotes resistance in the resident population of *L. reuteri* (Di Rienzi et al. 2018). Altogether, Lactobacilli seem to be protected from the toxic effects of antimicrobial long-chain FFAs in the gut, and they are capable of developing resistance towards these compounds. Since FFA-tolerant mutants were readily isolated *in vitro* for *L. monocytogenes* (described above), it is tempting to speculate that intestinal pathogens may develop resistance towards antimicrobial long-chain FFAs in the gut as well.

### Long-chain FFAs with antibiofilm activity against *L. monocytogenes*

Below growth-inhibitory concentrations, FFAs are known to selectively inhibit or disrupt biofilm formation by various pathogens (Kumar et al. 2020). *Listeria monocytogenes* forms disinfectant-tolerant biofilms on a variety of surfaces in food production environments. Consequently, biofilms are of major concern to the food industry since they can lead to contamination of food products (Mazaheri et al. 2021, Osek et al. 2022). A high-throughput screen for antibiofilm agents identified saturated medium- and long-chain FFAs ranging from C9 to C14 as effective inhibitors of *L. monocytogenes* biofilm development, while longer saturated FFAs (C16–C18) had stimulatory effects on biofilm formation (Nguyen et al. 2012). The concentrations used for testing antibiofilm activity did not change planktonic cell density, suggesting that saturated medium- and long-chain FFAs act as signaling molecules to modulate biological pathways involved in biofilm formation. Their antibiofilm targets in *L. monocytogenes* are presently unknown, but in Gram-negative bacteria, saturated FFAs are known to suppress quorum sensing signaling pathways controlling biofilm formation (Santhakumari et al. 2017, Kumar et al. 2020). Notably, the study by Nguyen et al. (2012) did not examine the effect of unsaturated medium- and long-chain FFAs on the formation of biofilms by *L. monocytogenes*. Furthermore, the study focused on single-species *L. monocytogenes* biofilms, which are considered more sensitive to disinfectants and antimicrobials relative to the mixed-species biofilms commonly found in the food industry (Yuan et al. 2020). Uncovering the biofilm-inhibitory properties of saturated and unsaturated FFAs may support the future development of new antibiofilm strategies against *L. monocytogenes* in single- and/or mixed-species biofilms.

### FFAs inhibit the activity of key virulence regulator PrfA in *L. monocytogenes*

**Figure 5.**
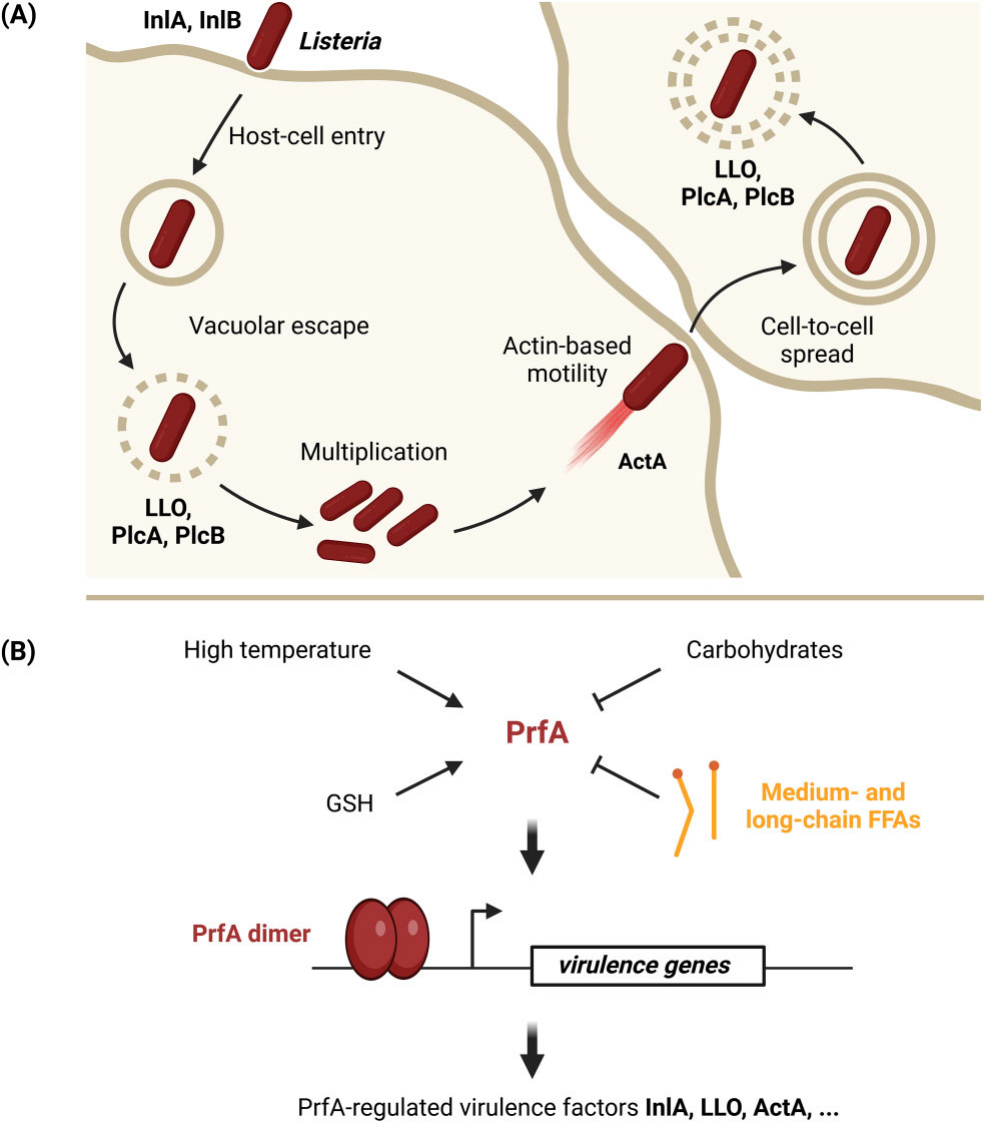
Medium- and long-chain FFAs inhibit PrfA-mediated expression of virulence factors required for the intracellular lifestyle of *L. monocytogenes*. (A) The internalins InlA and InlB promote bacterial entry into nonphagocytic host cells. The pore-forming toxin LLO, and the phospholipases PlcA and PlcB, enable bacterial escape from the primary vacuole. Inside the cytosol, the pathogen multiplies and spreads to adjacent cells using the actin assembly-inducing protein ActA. Finally, LLO, PlcA, and PlcB promote bacterial escape from the secondary vacuole formed upon entry of *L. monocytogenes* into the neighboring cell. (B) The transcriptional regulator PrfA activates transcription of virulence genes required for the intracellular lifestyle of *L. monocytogenes*. The level or activity of PrfA is affected by various signals from the environment, such as high temperature, carbohydrates, and glutathione (GSH). Furthermore, exposure to medium-chain saturated and long-chain unsaturated FFAs prevent PrfA from activating transcription of virulence genes encoding InlA, LLO, ActA, and so on.

**Figure 6.**
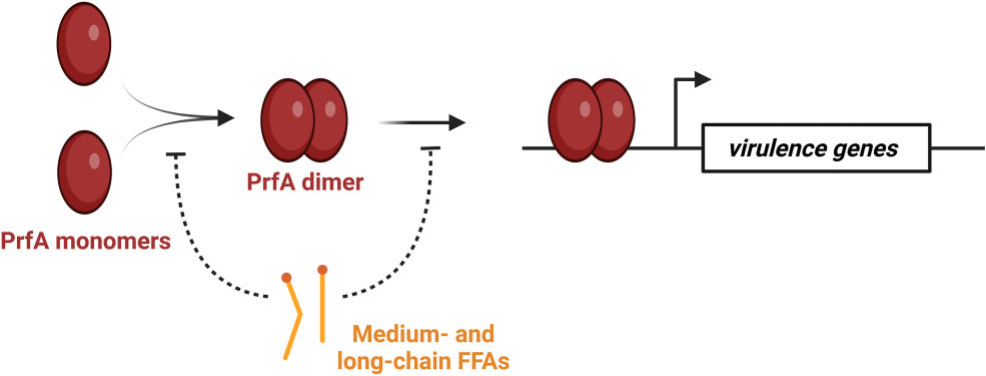
Model of how medium- and long-chain FFAs interfere with the virulence regulator PrfA. Specific medium-chain saturated and long-chain unsaturated FFAs are known to prevent PrfA from binding to DNA. Furthermore, exposure to FFAs inhibit PrfA-dependent activation of virulence genes in *L. monocytogenes*. These observations support a model where specific medium- and long-chain FFAs inhibit the dimerization and/or DNA-binding activity of PrfA.

Low levels of medium- and long-chain FFAs are known to reduce expression of virulence genes essential for the intracellular lifestyle of *L. monocytogenes* (Sternkopf Lillebaek et al. 2017, Dos Santos et al. 2020). During infection, *L. monocytogenes* enters and multiplies within a variety of host cells, and the pathogen spreads from cell-to-cell through a mechanism that involves actin polymerization (Freitag et al. 2009, Koopmans et al. 2023) (Fig. 5A). Several virulence factors contribute to the intracellular lifestyle of *L. monocytogenes*, including a pore-forming toxin, LLO, and ActA, which promotes actin polymerization and cell-to-cell spread. The transcription regulator PrfA is essential for the expression of genes encoding LLO, ActA, and other key virulence factors (Scortti et al. 2007). Various signals from the environment are known to affect the level or activity of PrfA, such as temperature changes, the presence of readily utilizable carbohydrates, bacterial- and host-derived glutathione (GSH), or other peptides (Johansson and Freitag 2019, Tiensuu et al. 2019) (Fig. 5B). Curiously, exposure to subinhibitory levels of medium-chain and long-chain FFAs generates an inhibitory signal that prevents PrfA-dependent activation of virulence gene transcription (Sternkopf Lillebaek et al. 2017, Dos Santos et al. 2020). The antivirulence activity of medium- and long-chain FFAs seems to rely on the length of the carbon chain and degree of unsaturation (Dos Santos et al. 2020). Among the saturated FFAs, lauric acid (C12:0) and myristic acid (C14:0) exert PrfA-inhibitory activity, whereas palmitic acid (C16:0) and stearic acid (C18:0) have no effect on virulence gene expression. Exposure to long-chain unsaturated FFAs, such as eicosapentaenoic acid (C20:5), linoleic acid (C18:2), and γ-linolenic acid (C18:3) (Table S1, Supporting Information), leads to repression of PrfA-dependent transcription as well (Sternkopf Lillebaek et al. 2017, Dos Santos et al. 2020) (Fig. 5B). Importantly, the PrfA-inhibitory activity of a specific FFA correlates well with its ability to prevent PrfA from binding to DNA. These findings support a model where long-chain unsaturated FFAs and medium-chain saturated FFAs interfere directly with the DNA-binding activity of PrfA (Fig. 6). Some FFAs act as antivirulent agents only [e.g. myristic acid (C14:0) and oleic acid (18:1)], whereas others exert a dual inhibitory effect: at low (subinhibitory) doses, they show antivirulent activity, whereas at high doses, they prevent bacterial growth [e.g. palmitoleic acid (C16:1) and lauric acid (C12:0)]. Importantly, mutant strains that grow in the presence of high levels of antimicrobial medium- and long-chain FFAs remain susceptible to the PrfA-inhibitory activity of FFAs (Thomasen et al. 2022a, b). Thus, although *L. monocytogenes* becomes resistant to the antimicrobial activity of FFAs, it can still be targeted by their antivirulent activity.

Transcriptomic analyses of *L. monocytogenes* exposed to various FFAs confirmed that specific medium- and long-chain FFAs act to down-regulate the expression of PrfA-dependent virulence genes (Chen et al. 2021, Thomasen et al. 2022b). Exposure to a subinhibitory level of lauric acid (C12:0) repressed the expression of three genes (*plcA, hly*, and *actA*) belonging to the PrfA regulon, whereas only a single gene, encoding the cochaperone GroES, was significantly up-regulated after lauric acid-exposure (Thomasen et al. 2022b). Similarly, treatments with MIC levels of long-chain unsaturated FFAs led to repression of PrfA-dependent virulence genes (Chen et al. 2021). Notably, up to 50% of the coding genes in *L. monocytogenes* were differentially expressed upon exposure to growth-inhibitory levels of palmitoleic acid (C16:1), linoleic acid (C18:2), and α-linolenic acid (C18:3) (Chen et al. 2021). Key genes associated with the response to acid, bile, osmotic, and oxidative stress were found to be downregulated in response to antimicrobial FFAs. In *L. monocytogenes*, stress response genes are primarily regulated by the alternative sigma factor Sigma B, which also controls the expression of *prfA* and other virulence-associated genes contributing to adaptation during the intestinal phase of infection (Tiensuu et al. 2019). Importantly, Sigma B is not required for growth in the presence of the antimicrobial FFAs palmitoleic acid (C16:1) and γ-linoleic acid (C18:3) (Sternkopf Lillebaek et al. 2017). Altogether, these findings suggest that exposure to antimicrobial FFAs does not activate the Sigma B-dependent stress response in *L. monocytogenes*. In contrast, antimicrobial FFAs appear to downregulate many genes involved in the adaptation to stressful conditions encountered in the gastrointestinal tract, including acid, bile, and osmotic stress (Chen et al. 2021). Intriguingly, a Δ*prfA* mutant strain can grow at high concentrations of medium- and long-chain FFAs, suggesting that unknown regulatory activities of PrfA increases the sensitivity of *L. monocytogenes* toward antimicrobial FFAs (Sternkopf Lillebaek et al. 2017). Indeed, in addition to the PrfA-dependent virulence genes described above, PrfA is known to control the expression of multiple genes involved in stress tolerance and general metabolism (Milohanic et al. 2003, Marr et al. 2006, Henderson et al. 2020). Clearly, PrfA plays a central role in the response of *L. monocytogenes* against antimicrobial and antivirulent medium- and long-chain FFAs (Sternkopf Lillebaek et al. 2017, Dos Santos et al. 2020).

Considering the inhibitory effects of specific medium- and long-chain FFAs against *L. monocytogenes*, they could have a protective effect towards infections by this pathogen. Cell-based infection models are commonly used to evaluate the ability of *L. monocytogenes* to invade host cells, multiply within them, and spread to the neighboring cells. The Caco-2 enterocyte-like cell-line has been used to examine how specific FFAs derived from milk affect the invasiveness of *L. monocytogenes* (Petrone et al. 1998). A total of 12 short-, medium-, and long-chain FFAs were tested for their ability to protect Caco-2 from invasion by *L. monocytogenes*. The FFA levels used in this experiment did not affect the growth and survival of bacteria and Caco-2 cells, however, it should be noted that long-chain FFAs are known to upregulate the expression of E-cadherin, which serves as target for the listerial virulence factor InlA (described below) (Jiang et al. 1995). After 1 hour of FFA-exposure, the bacterial invasion of FFA-treated cells was reduced by 20- to 500-fold compared with untreated cells (Petrone et al. 1998). Among the FFAs tested, the unsaturated long-chain FFA linolenic acid (C18:3) had the most potent effect on invasion. In line with this, C18:3 exposure efficiently down-regulates expression of virulence genes in *L. monocytogenes*, including PrfA-dependent transcription of *inlA* encoding the surface protein InlA, which is required for invasion of Caco-2 cells (Fig. 5A) (Sternkopf Lillebaek et al. 2017, Dos Santos et al. 2020). Other FFAs with known PrfA-inhibitory activity, such as lauric acid (C12:0), oleic acid (C18:1), and linoleic acid (C18:2), are potent inhibitors of bacterial invasion as well (Petrone et al. 1998). The saturated long-chain FFA stearic acid (C18:0) is the least effective in preventing invasion by *L. monocytogenes* and this FFA does not affect the expression of PrfA-activated virulence genes, including *inlA* (Sternkopf Lillebaek et al. 2017, Dos Santos et al. 2020). These findings suggest that FFAs with PrfA-inhibitory activity are interesting candidates for antivirulence compounds acting to prevent *L. monocytogenes* from invading enterocytes.

### Fatty acid biosynthesis plays a role in the virulence of *L. monocytogenes*

The membrane phospholipids of *L. monocytogenes* are mainly composed of branched-chain fatty acids, which are synthesized by the FASII system (Sauer et al. 2019). *Listeria monocytogenes* encodes putative homologs of the core enzymes for bacterial type II fatty acid synthesis, including the β-ketoacyl-acyl carrier protein synthase III (FabH), which carries out the first condensation reaction in fatty acid biosynthesis (Singh et al. 2009). The FabH enzyme in *L. monocytogenes* exhibits selectivity for anteiso branched-chain fatty acid precursors, which results in a membrane enriched in this type of fatty acid. Importantly, a high content of anteiso branched-chain fatty acids increases membrane fluidity, which contributes to *L. monocytogenes* stress resistance and plays a role in bacterial pathogenesis (Sun and O’Riordan 2010). Anteiso branched-chain fatty acids enhance the resistance of *L. monocytogenes* toward macrophage killing by protecting against the antimicrobial peptides and peptidoglycan hydrolases produced in the phagosome (Sun et al. 2012). Furthermore, conditions that support a high content of anteiso branched-chain fatty acids promote the production of LLO, suggesting that changes in membrane fluidity could alter the activity of PrfA (Sun et al. 2012). The molecular mechanisms underlying the effect of membrane fatty acid composition on LLO production remains to be determined.

The enoyl-acyl carrier protein reductase I (FabI) plays a role in the elongation cycle of fatty acid synthesis (Yao et al. 2016). Inhibition of FabI, using a selective FabI inhibitor, prevents endogenous fatty acid synthesis by 80% and lowers growth of *L. monocytogenes* in laboratory medium (Yao et al. 2016). In the presence of a FabI inhibitor, supplementation with exogenous fatty acids partially restores normal growth in laboratory medium, and the exogenous fatty acids are incorporated into the membrane phospholipids. Importantly, inhibition of FabI during intracellular infection of host cells has detrimental effects on *L. monocytogenes*, which cannot be reverted by the acquisition of host cell fatty acids (Yao et al. 2016). Clearly, FabI represents an interesting target for drugs acting against intracellular *L. monocytogenes*.

### Exogenous unsaturated long-chain FFAs promote growth by *L. monocytogenes* at low temperature

*Listeria monocytogenes* is a psychrotrophic bacterium holding the ability to alter the composition of its membrane to sustain optimum membrane fluidity at low temperature (Annous et al. 1997). A high content in the membrane of anteiso branched-chain fatty acids is known to be a critical factor for growth of *L. monocytogenes* at refrigeration temperatures (Annous et al. 1997, Singh et al. 2009). When the temperature declines, *L. monocytogenes* responds by rising the anteiso-C15:0 fatty acid content in the membrane to maintain optimal membrane fluidity (Annous et al. 1997, Zhu et al. 2005).

Recent studies suggest that exogenous long-chain fatty acids are incorporated in the membrane to promote growth at low temperatures (Flegler et al. 2022, Touche et al. 2023). *Listeria monocytogenes* encodes homologs of the fatty acid kinase system FakA/FakB, which allows the bacterium to utilize exogenous FFA as precursors for membrane building blocks (Yao et al. 2016, Yao and Rock 2017). In Gram-positive bacteria, the fatty acid kinase system binds and phosphorylates exogenous FFAs taken up by the cells. The resulting acyl-phosphates then enters the phospholipid synthesis pathway, and the phospholipids are finally incorporated in the bacterial membrane (Yao and Rock 2017). Growth medium supplemented with unsaturated long-chain FFAs induces an increase of the growth rate of *L. monocytogenes* at 5°C–6°C, whereas saturated long-chain FFAs inhibit bacterial growth or leave it unaffected at low temperatures (Flegler et al. 2022, Touche et al. 2023). Incorporation of unsaturated FFAs decreases the phospholipid melting point temperature, which allows *L. monocytogenes* to compensate for the decrease in membrane fluidity caused by low temperatures. Notably, *L. monocytogenes* does not encode a functional fatty acid β-oxidation pathway, which means that exogenous long-chain FFAs do not serve as an energy source for *L. monocytogenes* (Glaser et al. 2001, Sauer et al. 2019). Thus, exogenous unsaturated long-chain FFAs seem to be used exclusively as membrane building blocks to promote the fitness of *L. monocytogenes* under stressful conditions, such as low temperature.

### Dietary fat affects the course of *L. monocytogenes* infection

The effect of different dietary fat on *L. monocytogenes* infection has been evaluated using various animal infection models (Harrison et al. 2013, Las Heras et al. 2019). Several studies have shown that mice fed with fish oil are more susceptible to infection by *L. monocytogenes* relative to mice fed on a low-fat diet (de Pablo et al. 2000, Puertollano et al. 2004). A diet containing fish oil impairs the clearance of *L. monocytogenes* from the organs of mice, and survival of the infected animals is significantly reduced. The polyunsaturated long-chain fatty acids in fish oil are known to hold anti-inflammatory and immunomodulatory properties, which may reduce the cellular host defense against systemic infections caused by intracellular pathogens, including *L. monocytogenes* (Harrison et al. 2013, Husson et al. 2016). When infected with *L. monocytogenes*, the survival of animals fed diets containing olive oil rich in monounsaturated oleic acid (C18:1), or hydrogenated coconut oil rich in saturated lauric acid (C12:0), is higher than the survival of mice fed with fish oil (de Pablo et al. 2000). Furthermore, dietary supplementation with CLA for up to 4 weeks does not affect the resistance to *L. monocytogenes* in mice (Turnock et al. 2001). Notably, the mouse experiments were performed by intravenous or intraperitoneal injection of *L. monocytogenes*, leaving out the gastrointestinal stage of infection (de Pablo et al. 2000, Turnock et al. 2001, Puertollano et al. 2004, Harrison et al. 2013). Thus, the effect of CLA and dietary oils rich in unsaturated long-chain fatty acids should be reassessed using a model that allows evaluation of the gastrointestinal stage of infection by *L. monocytogenes*.

In mice, infection by *L. monocytogenes* via the intestinal route is relatively low due to a poor interaction between the internalin InlA and the murine receptor E-cadherin (Lecuit et al. 1999). Notably, mice can be infected perorally using the strain *L. monocytogenes* EGDe^m^ expressing a modified InlA, which interacts with the murine E-cad receptor (Wollert et al. 2007, Monk et al. 2010). This strain was used in a recent study investigating the influence of a high-fat westernized diet, rich in saturated fat, on host susceptibility to infection by *L. monocytogenes* via the intestinal route (Las Heras et al. 2019). The animals consumed either a high-fat diet, a low-fat diet, or regular chow, for 2 weeks. Then, the mice were infected perorally with *L. monocytogenes*, and infection was allowed to progress for 3 days. Mice fed on westernized high-fat diet were more susceptible to infection compared to low-fat- or chow-fed animals. The bacterial burden in the spleen, caecum, and lymph nodes was significantly higher in animals fed with high-fat diet compared to chow-fed diet. Furthermore, profound changes in the host response and gut microbiota could be observed both pre- and postinfection. Altogether, this study concludes that consumption of a westernized high-fat diet is a significant factor influencing host resistance to *L. monocytogenes* infection (Las Heras et al. 2019). Notably, the authors did not examine the potential effects of a high-fat westernized diet on listerial expression of virulence factors.

Another study aimed to determine how high intake of milk fat affects intestinal colonization by *L. monocytogenes* in rats (Sprong et al. 1999). After adaptation to either low milk fat or high milk fat diets, rats were infected orally with *L. monocytogenes*. Interestingly, greater milk fat consumption inhibits colonization of this pathogen and reduces diarrhea in infected animals. The gastric contents of rats displayed an enhanced antilisterial activity with the amount of fat consumed. When analyzing the composition of the gastric contents, higher concentrations of FFAs were observed when larger amounts of dietary fat were consumed (Sprong et al. 1999). The antilisterial activity was primarily observed in the stomach, whereas no killing of *L. monocytogenes* was found in the contents of the small and large intestine of rats. These observations suggest that milk fat-mediated killing of *L. monocytogenes* most likely takes place in the gastric lumen. Indeed, various FFAs with known antibacterial activity against *L. monocytogenes* is found at higher concentrations in the stomach of rats fed on high-fat milk, including lauric acid (C12:0). Although no antibacterial activity could be observed, specific medium- and long-chain FFAs with known PrfA-inhibitory activity are present in higher levels in the small intestine of rats fed on a high-fat milk (Sprong et al. 1999). It is, therefore, tempting to speculate that specific FFAs derived from dietary fat could act as antivirulence agents in the small intestine, where *L. monocytogenes* is expected to initiate its infection.

## Concluding remarks

Foodborne pathogens encounter a variety of long-chain FFAs in the gastrointestinal tract, and multiple studies have confirmed their potential as antimicrobial and/or antivirulence agents. The consumption of dietary fat affects the course of infection, however, for most enteric pathogens the effect of dietary supplementation of specific long-chain FFAs with antimicrobial and/or antivirulent activities remains to be evaluated. The dietary fat type is expected to affect the complex interplay between the host response, the gut microbiota, and the pathogen at the gastrointestinal stage of infection (Caesar et al. 2015, Chadaideh and Carmody 2021). Furthermore, the dose and timing of fatty acid intake may determine the outcome of pathogen exposure (Husson et al. 2016). Thus, more studies are required to systematically explore the impact of different types and doses of long-chain FFAs in models that closely simulate the infectious disease in humans caused by foodborne pathogens.

Although naturally occurring long-chain FFAs show antivirulence activity *in vitro*, they may not be the best candidates for antivirulence therapy. FFAs serve as substrates for metabolic activities by the host and resident microbiota in the gastrointestinal tract, and the concentrations required to reduce infection appear to be relatively high. However, detailed information on how inhibitory FFAs interact with regulatory proteins may be used as inspiration to develop more potent antivirulence therapeutics. Indeed, structural information on how palmitoleic acid (C16:1) interacts with ToxT allowed the design of a new class of ToxT inhibitors that prevents virulence gene expression more efficiently than naturally occurring FFAs (Woodbrey et al. 2017). More specifically, the folded conformation of the monounsaturated long-chain FFA found in the crystal structure of ToxT served as inspiration for designing more effective ToxT inhibitors, and further improvements of their inhibitory potential resulted in a second-generation indole derivative with decreased cytotoxicity that protects against *V. cholerae* colonization in a mouse model (Woodbrey et al. 2018). Similar structure–function based approaches may lead to the development of novel anti-infective compounds targeting key virulence regulators in other bacterial pathogens.

In the past, the antimicrobial potency of FFAs has been studied very intensively to identify the FFAs with the greatest efficacy against bacterial pathogens. Although foodborne pathogens and members of the gut microbiota may develop resistance toward the antimicrobial actions of FFAs, their role as antivirulence agents should be further investigated. Detailed knowledge on how long-chain FFAs interfere with bacterial virulence may stimulate the development of novel anti-infective strategies in the future.

## Supplementary Material

fuad037_Supplemental_File
