# Supplementary material for: Anti-infective activities of long-chain fatty acids against foodborne pathogens
Source: FEMS Microbiol Rev. 2023 Jul 12;47(4):fuad037. doi: 10.1093/femsre/fuad037 (PMC10368373; doi:10.1093/femsre/fuad037)
Supplement: fuad037_Supplemental_File [file fuad037_supplemental_file.docx]

**Supplementary Table 1.** Chemical structures of medium- and long-chain free fatty acids (FFAs) mentioned in this review.

| **FFAs** | **Chemical structure** | **Double bond position and orientation** |
| --- | --- | --- |
| **Medium-chain FFAs** | | |
| Caprylic acid  (C8:0) | 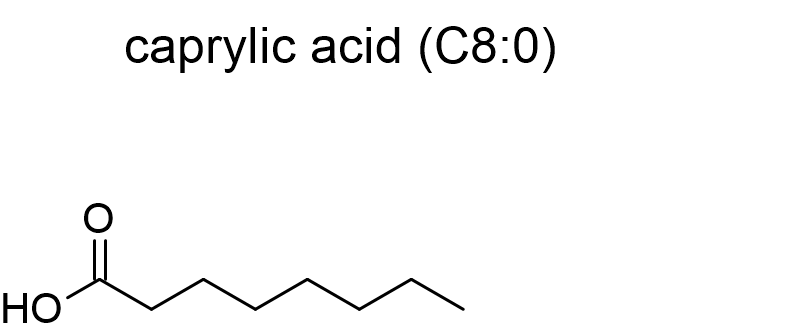 | - |
| Decanoic acid  (C10:0) | 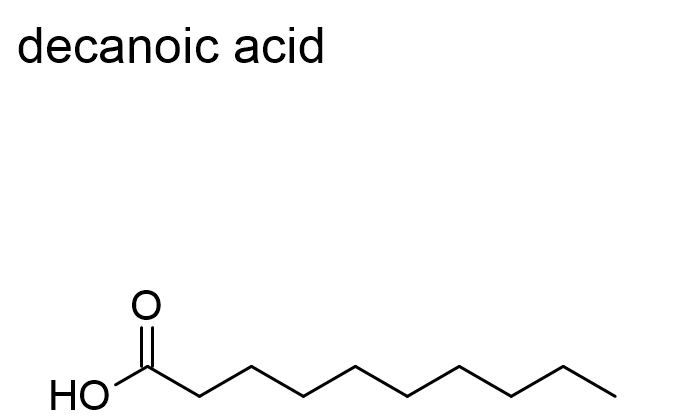 | - |
| Lauric acid  (C12:0) | 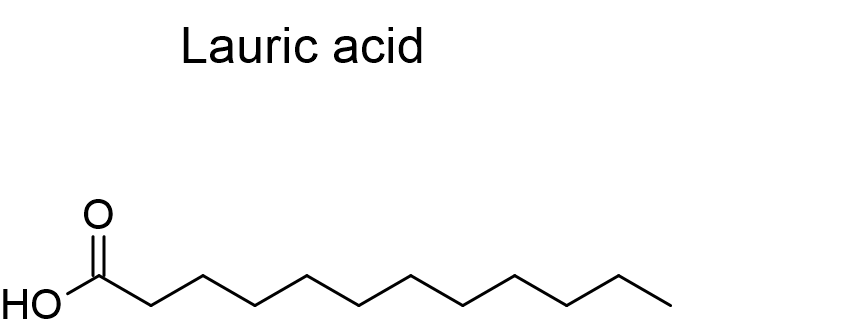 | - |
| **Long-chain FFAs** | | |
| Myristic acid  (C14:0) | 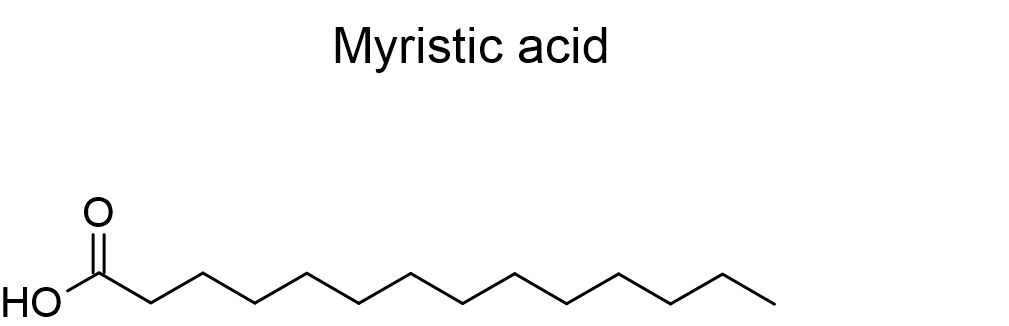 | - |
| Palmitic acid  (C16:0) | 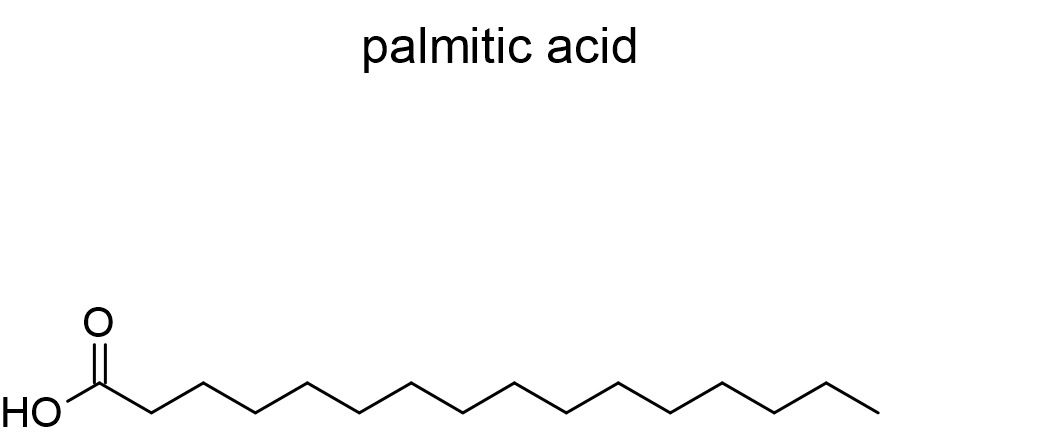 | - |
| Palmitoleic acid  (C16:1) | 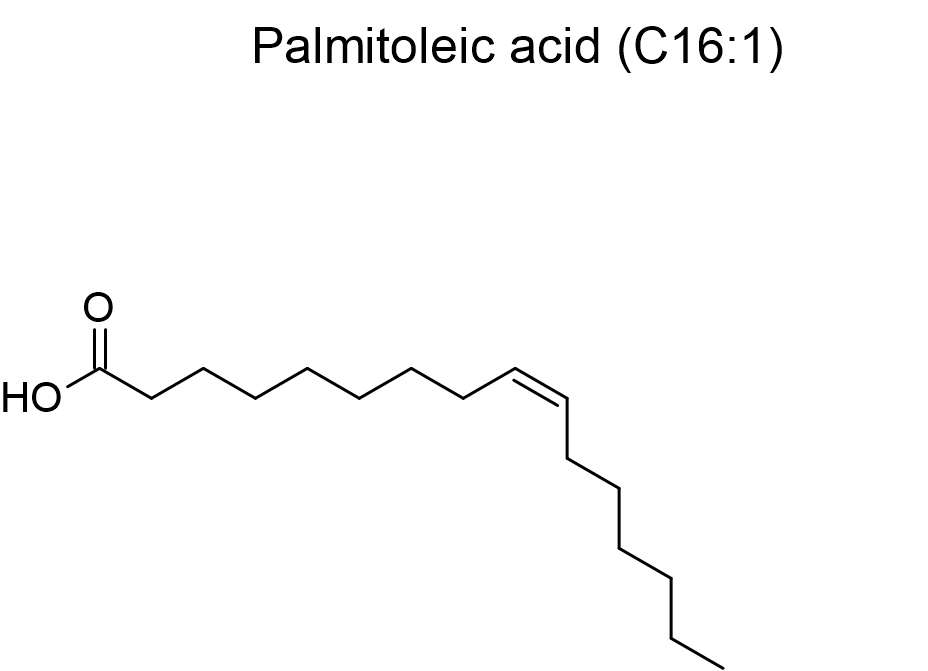 | *cis*-9 |
| Diffusible signal factor (DSF) family member:  *cis*-2-hexadecenoic acid (c2-HDA)  (C16:1) | 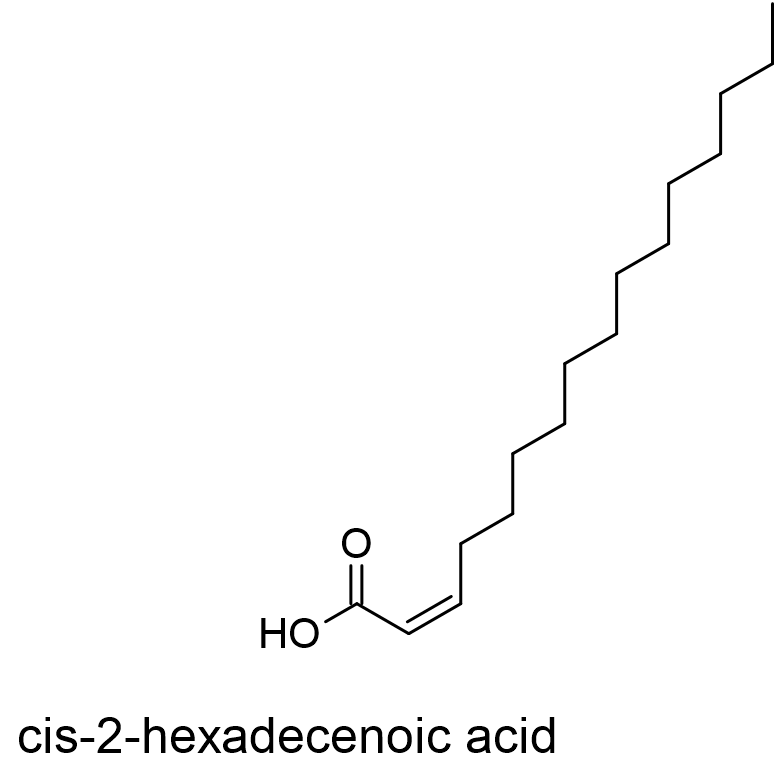 | *cis*-2 |
| Stearic acid  (C18:0) | 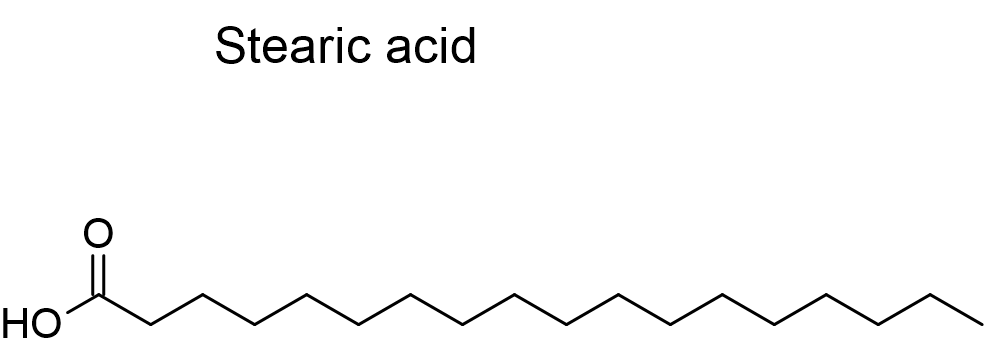 | - |
| Oleic acid  (C18:1) | 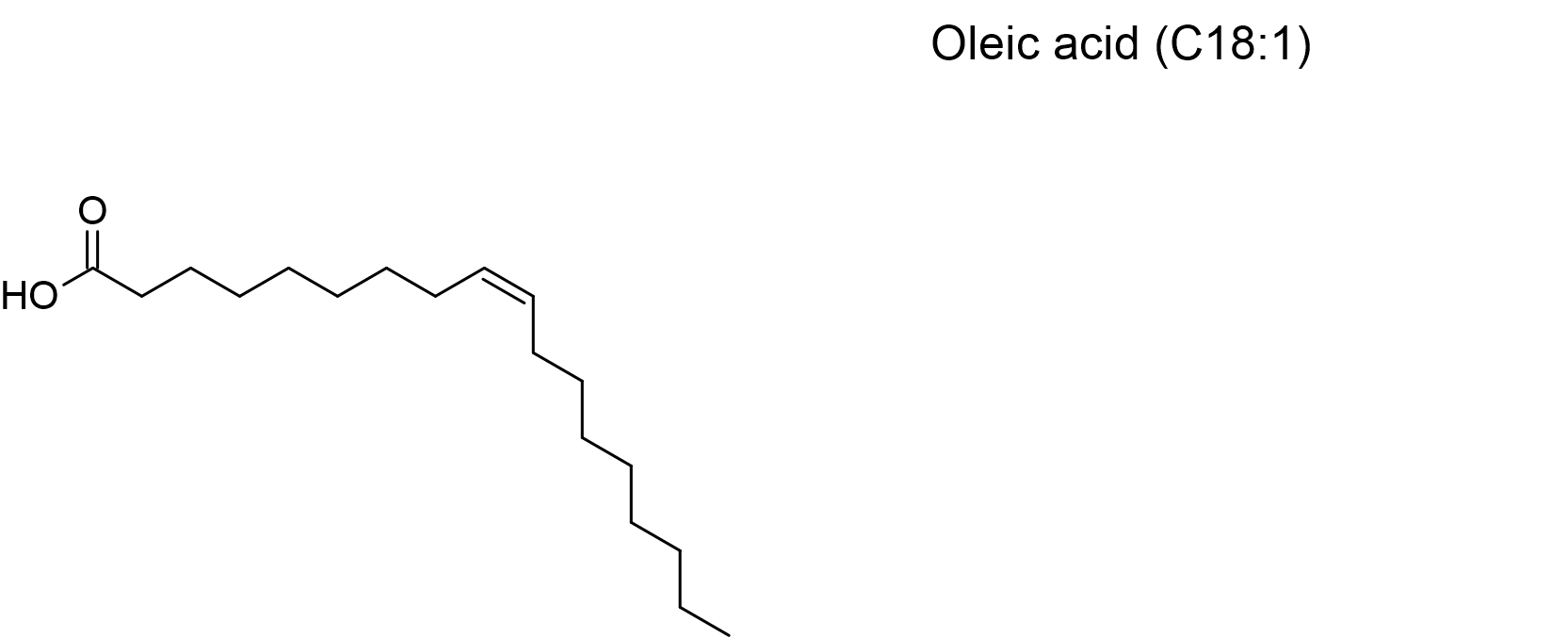 | *cis*-9 |
| Linoleic acid  (C18:2) | 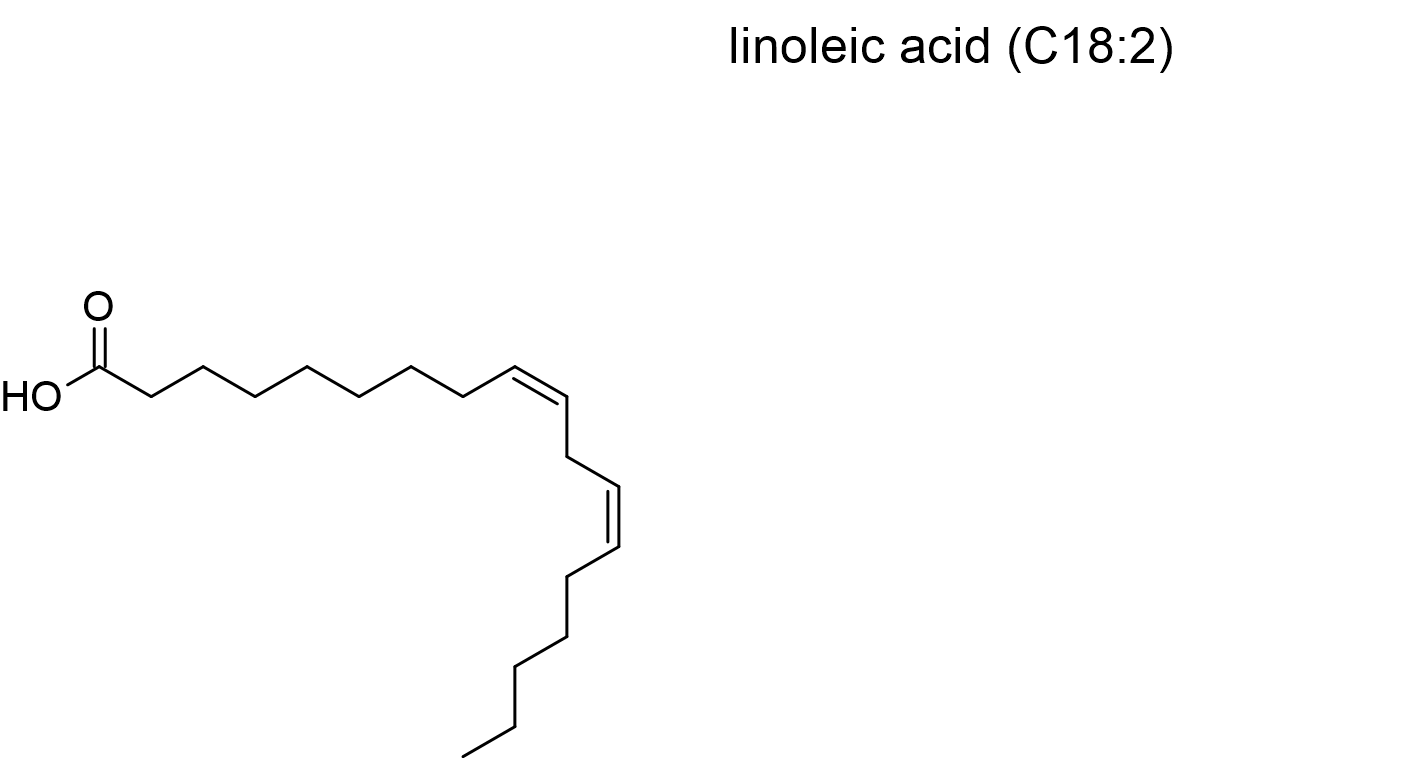 | *cis*-9,  *cis*-12 |
| Conjugated linoleic acid (CLA).  Example: Rumenic acid  (C18:2) | 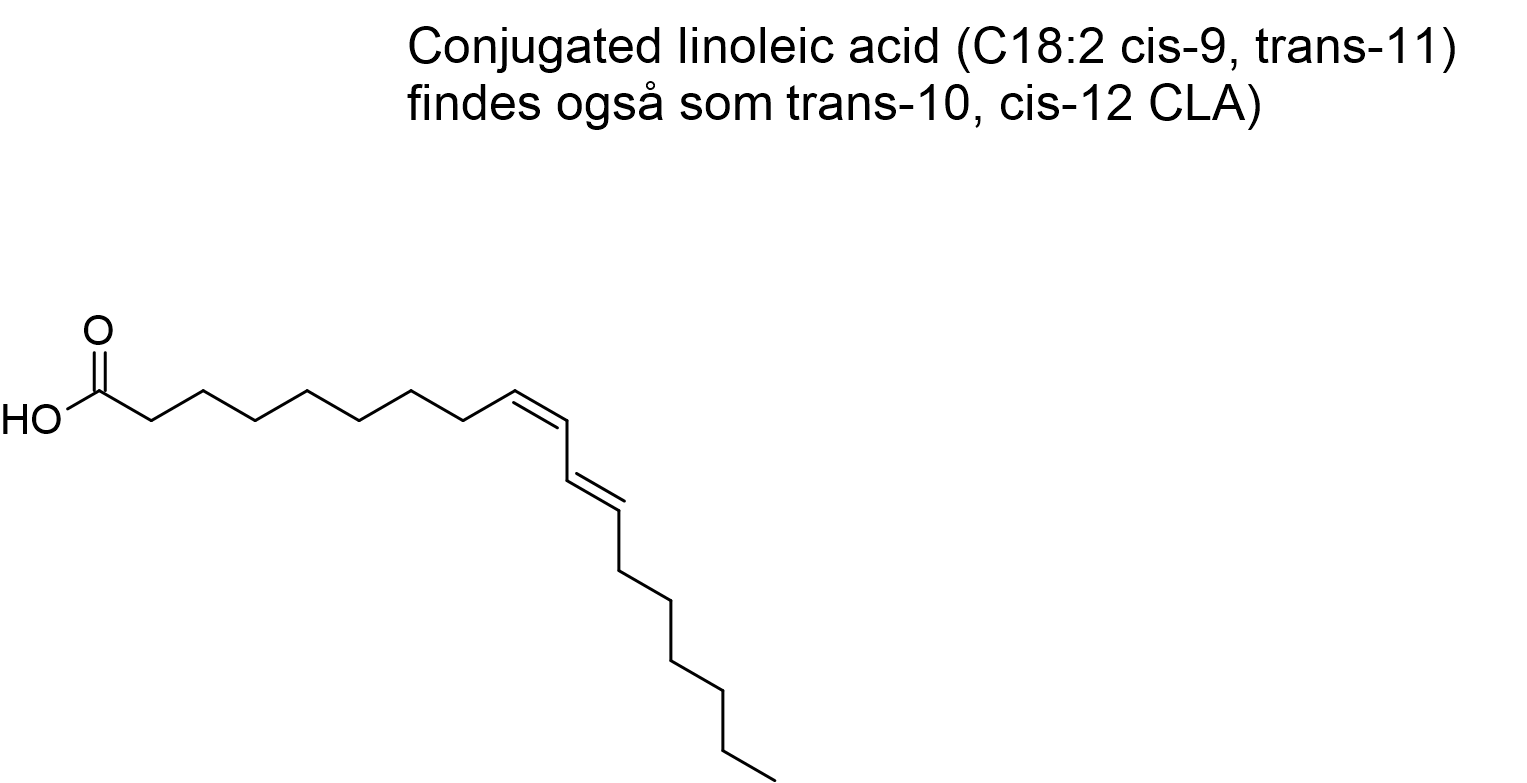 | *cis*-9,  *trans*-11 |
| α-linolenic acid  (C18:3) | 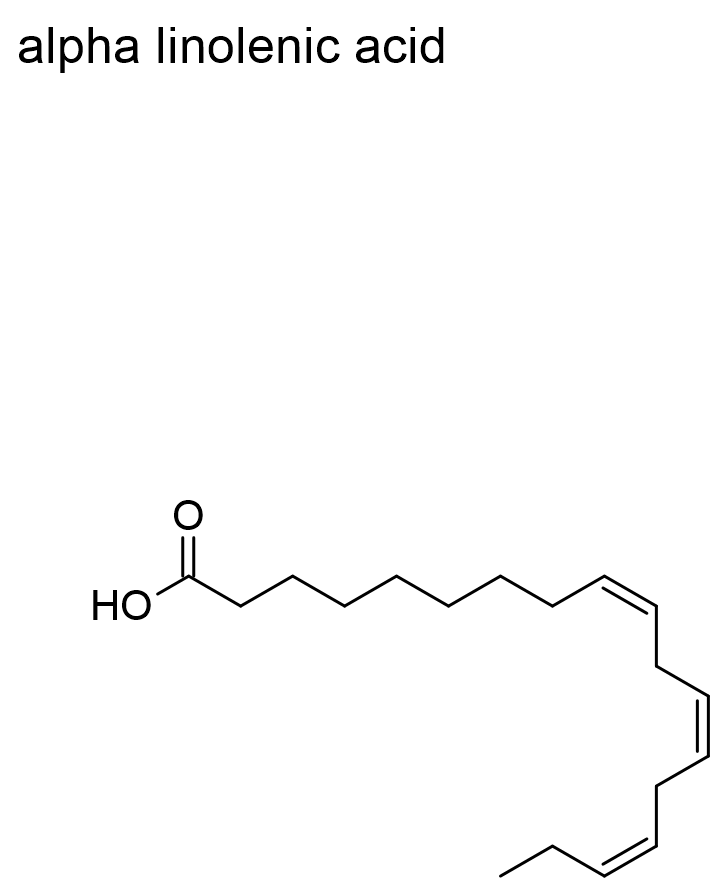 | *cis*-9,  *cis*-12,  *cis*-15 |
| γ-linolenic acid  (C18:3) | 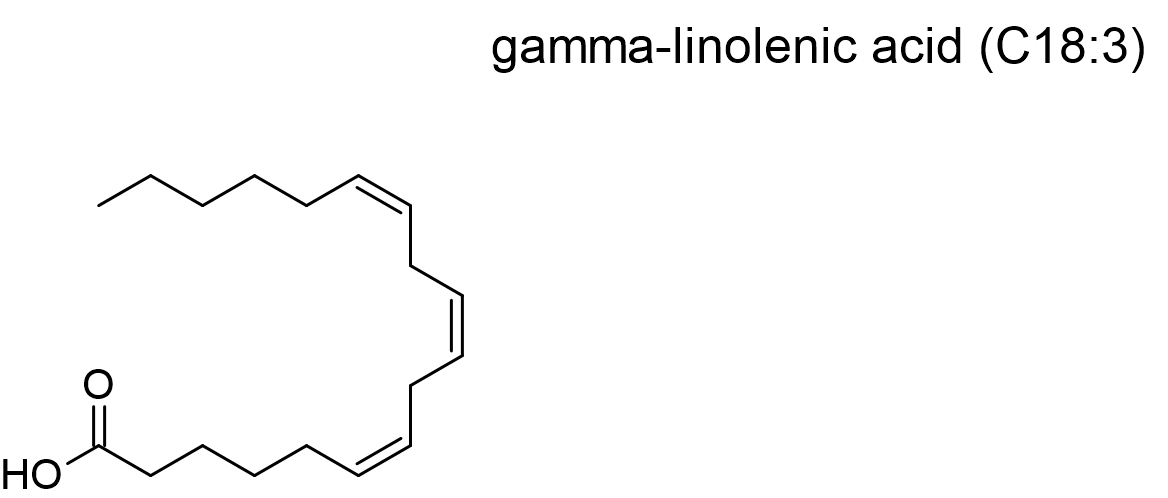 | *cis*-6,  *cis*-9,  *cis*-12 |
| Arachidonic acid  (C20:4) | 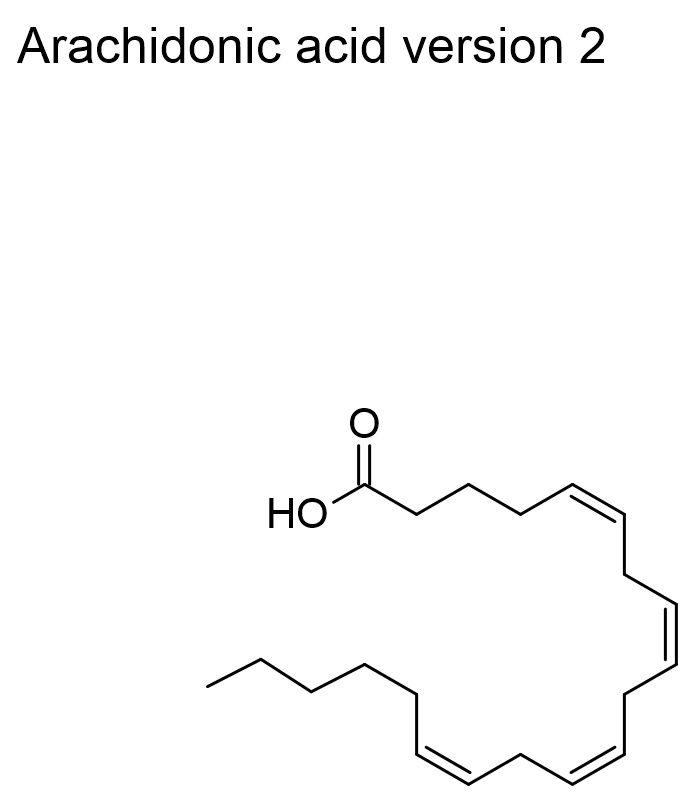 | *cis*-5,  *cis*-8,  *cis*-11,  *cis*-14 |
| Eicosapentaenoic acid  (C20:5) | 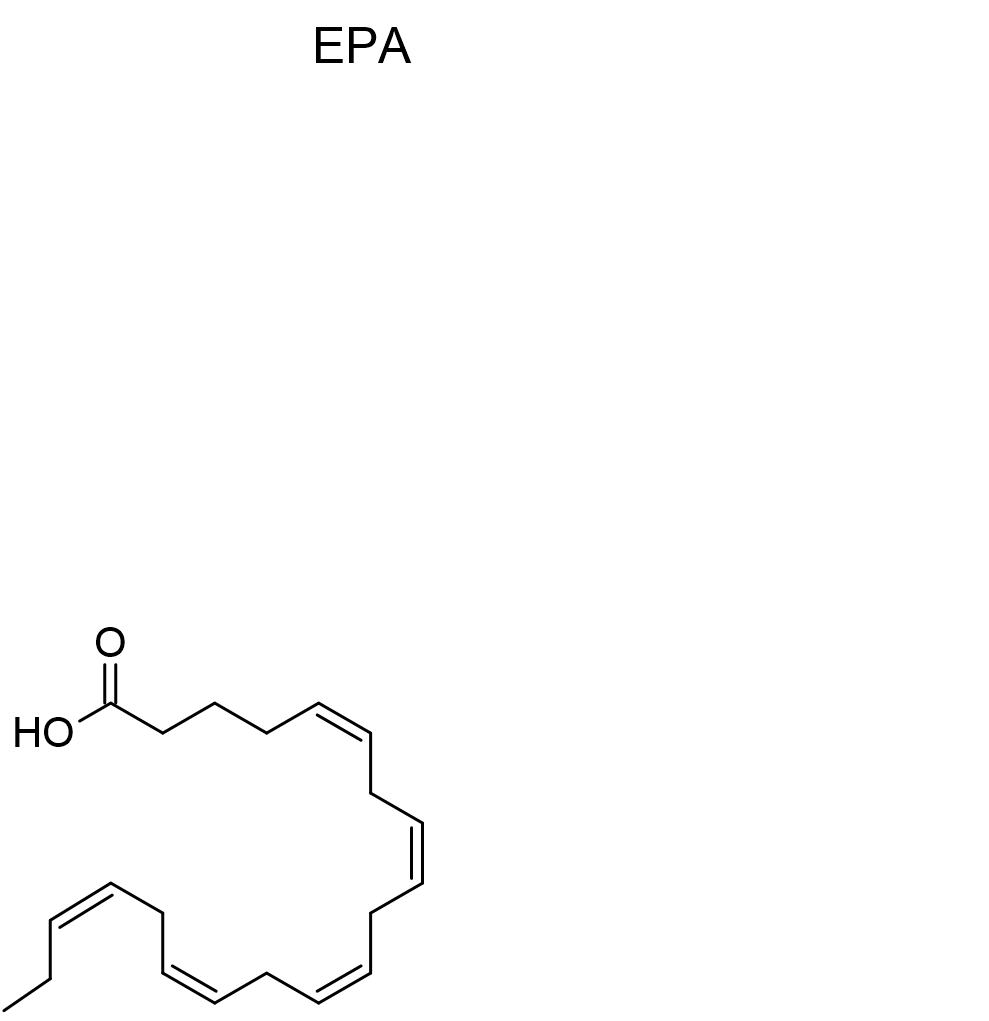 | *cis*-5,  *cis*-8,  *cis*-11,  *cis*-14,  *cis*-17 |
| Docosahexaenoic acid  (C22:6) | 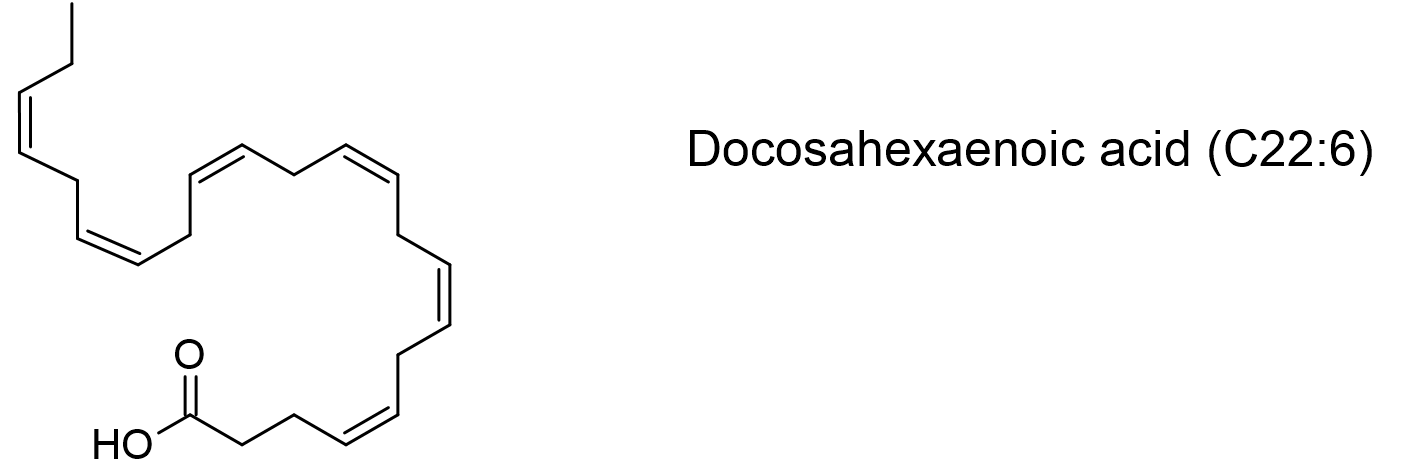 | *cis*-4,  *cis*-7,  *cis*-10,  *cis*-13,  *cis*-16,  *cis*-19 |
